# Inconsistencies within the proposed framework for stabilizing fungal nomenclature risk further confusion

**DOI:** 10.1128/jcm.01570-23

**Published:** 2024-03-05

**Authors:** Sarah E. Kidd, Ferry Hagen, Catriona L. Halliday, Alireza Abdolrasouli, Teun Boekhout, Pedro W. Crous, David H. Ellis, Juliet Elvy, Graeme N. Forrest, Marizeth Groenewald, Rosane C. Hahn, Jos Houbraken, Anderson M. Rodrigues, James Scott, Tania C. Sorrell, Richard C. Summerbell, Clement K. M. Tsui, Andrey Yurkov, Sharon C.-A. Chen

**Affiliations:** 1 National Mycology Reference Centre, SA Pathology, Adelaide, South Australia, Australia; 2 School of Biological Sciences, Faculty of Sciences, University of Adelaide, Adelaide, South Australia, Australia; 3 Westerdijk Fungal Biodiversity Institute, Utrecht, the Netherlands; 4 Institute for Biodiversity and Ecosystem Dynamics, University of Amsterdam, Amsterdam, the Netherlands; 5 Department of Medical Microbiology, University Medical Center Utrecht, Utrecht, the Netherlands; 6 Centre for Infectious Diseases and Microbiology Laboratory Services, Institute of Clinical Pathology and Medical Research, NSW Health Pathology, Westmead Hospital, Westmead, New South Wales, Australia; 7 Department of Medical Microbiology, King’s College Hospital, London, United Kingdom; 8 Department of Infectious Diseases, Imperial College London, London, United Kingdom; 9 College of Sciences, King Saud University, Riyadh, Saudi Arabia; 10 Department of Biochemistry, Genetics and Microbiology, Forestry and Agricultural Biotechnology Institute (FABI), University of Pretoria, Pretoria, South Africa; 11 Department of Biology, Molecular Microbiology, Utrecht University, Utrecht, the Netherlands; 12 Awanui Labs, Dunedin Hospital, Dunedin, New Zealand; 13 Rush University Medical Center, Chicago, Illinois, USA; 14 Medical Mycology Laboratory/Investigation, Faculty of Medicine, Federal University of Mato Grosso, Cuiabá, Mato Grosso, Brazil; 15 Júlio Muller Hospital, EBSERH, Cuiabá, Mato Grosso, Brazil; 16 Department of Microbiology, Immunology and Parasitology, Laboratory of Emerging Fungal Pathogens, Discipline of Cellular Biology, Federal University of São Paulo (UNIFESP), São Paulo, Brazil; 17 Dalla Lana School of Public Health, University of Toronto, Toronto, Ontario, Canada; 18 Sporometrics, Toronto, Ontario, Canada; 19 Sydney Infectious Diseases Institute, University of Sydney, Sydney, New South Wales, Australia; 20 Infectious Diseases Research Laboratory, National Center for Infectious Diseases, Singapore, Singapore; 21 Faculty of Medicine, University of British Columbia, Vancouver, Canada; 22 Leibniz Institute DSMZ—German Collection of Microorganisms and Cell Cultures, Braunschweig, Germany; Boston Children's Hospital, Boston, Massachusetts, USA

**Keywords:** taxonomy, nomenclature, medical mycology

## LETTER

We read with interest the recent publication by de Hoog and colleagues in the *Journal of Clinical Microbiology* (1) and support the goal to stabilize fungal nomenclature. Although we recognize the importance of stable naming, we offer comments on the need for clarity around the concepts introduced, consistency of the recommended nomenclature, process issues regarding the endorsement by professional groups and societies and the proposed oversight committee (working group), and the relationship between the proposed database and existing, officially recognized nomenclatural repositories.

We ask why the stability of nomenclature is more concerning for some pathogens (e.g., *Bipolaris*/*Curvularia australiensis*, *Emmonsia*/*Emergomyces crescens*) than others (e.g., *Ochroconis*/*Verruconis gallopava*, *Trichosporon*/*Apiotrichum mycotoxinovorans*), and question the consistency and clinical value of certain reporting recommendations; e.g., *Trichophyton violaceum* requiring the comment “member of *Trichophyton rubrum* complex,” when an analogous comment is not recommended for *Trichophyton indotineae*, or even for *Cryptococcus neoformans*/*C. gattii* and *Histoplasma capsulatum* (2, 3). The recommended option to continue using prior (now obsolete) *Candida* names appears inconsistent with the statement that the “[*Candida*] genus in the traditional sense is untenable.” Additionally, some nomenclature and reporting recommendations contradict previously published opinions (4–11), and we are concerned that recommending two names as alternatives for reporting will reverse the benefits of many years of advocacy for “One Fungus One Name” (12).

The transition to updated nomenclature has therapeutic relevance (13, 14) and is demonstrably manageable following published recommendations (7–10, 15) with the support of clinicians (16). Many clinical laboratories across Australasia and Europe have successfully implemented nomenclature change following recommendations under the guidance of local organizations, and some commercial identification system vendors (e.g., Bruker Biotyper) have implemented nomenclature updates in their databases. A reversal of this progress seems retrogressive and could erode trust in taxonomic science.

With the exception of being linked to a subscription resource, the proposed database (www.atlasclinicalfungi.org) appears similar to the officially recognized nomenclature repositories, MycoBank (www.mycobank.org), IndexFungorum (www.indexfungorum.org), and Fungal Names (www.nmdc.cn/fungalnames), housed within the Westerdijk Institute, Royal Botanic Gardens Kew, and the Institute of Microbiology of the Chinese Academy of Sciences, respectively, with all being listed as affiliations in de Hoog et al. (1). How will these databases be reconciled and managed to provide consistent messaging?

Eleven professional entities or organizations have endorsed the proposed framework, but the process of consultation was not delineated and did not include many members or stakeholders. How will this framework impact stakeholders outside of clinical mycology, particularly veterinary mycology (ISHAM being a society that also represents animal mycoses), but also biotechnology, food mycology, and agriculture? Given that some of the article’s authors (and us) are affiliated with the International Mycological Association Nomenclature Committee for Fungi, we suggest that they clarify the remit of the working group, and to what extent it can arbitrate on the use of validly published and approved fungal names. Furthermore, how do the editorial boards or committees for the endorsing organizations propose to work together to ensure publications and guidelines provide consistent messaging to avoid further confusion with regard to nomenclature?

We applaud the proposal to introduce a nomenclature working group under the auspices of ISHAM, but the group should draw upon expertise from diverse geographic areas and represent a platform for all stakeholders. Transparent appointment and decision-making processes with clear terms of reference will be required. Finally, although we have significant concerns about some of the proposals concerning fungal nomenclature, we certainly agree that it must be stabilized to optimize client-focused outcomes.
